# 2,2′-Diamino-*N*,*N*′-(*o*-phenyl­ene)di­benz­amide

**DOI:** 10.1107/S1600536809009283

**Published:** 2009-03-25

**Authors:** Irvin Booysen, Thomas I. A. Gerber, Eric Hosten, Peter Mayer

**Affiliations:** a Department of Chemistry, Nelson Mandela Metropolitan University, 6031 Port Elizabeth, South Africa; b Department of Chemistry, Ludiwig-Maximilians University, D-81377 München, Germany

## Abstract

In the structure of the title compound, C_20_H_18_N_4_O_2_, the N—H and C=O bonds are *trans* to each other and the amide O atoms are *syn* to the *ortho* amino N atom in the benzoyl rings. The amide groups form dihedral angles of 8.4 (2) and 13.8 (2)° with their respective benzoyl rings, and dihedral angles of 51.85 (16) and 51.19 (17)° with the phenyl­enediamine ring. In the crystal, a centrosymmetric dimer is formed by inter­molecular N—H⋯O hydrogen bonds, resulting in an *R*_2_^2^(14) descriptor on a unitary level of graph-set analysis, and three intramolecular N—H⋯O bonds also occur.

## Related literature

For the synthesis, see: Black & Rothnie (1983 ▶). For metal coordination, see: Booysen *et al.* (2008 ▶). For stereoselectivity in synthesis, see: Valik *et al.* (2002 ▶). For applications of polyamides, see: Kang *et al.* (2001 ▶). For related structures, see Gowda *et al.* (2003 ▶, 2008 ▶). For graph-set notation, see: Bernstein *et al.* (1995 ▶).
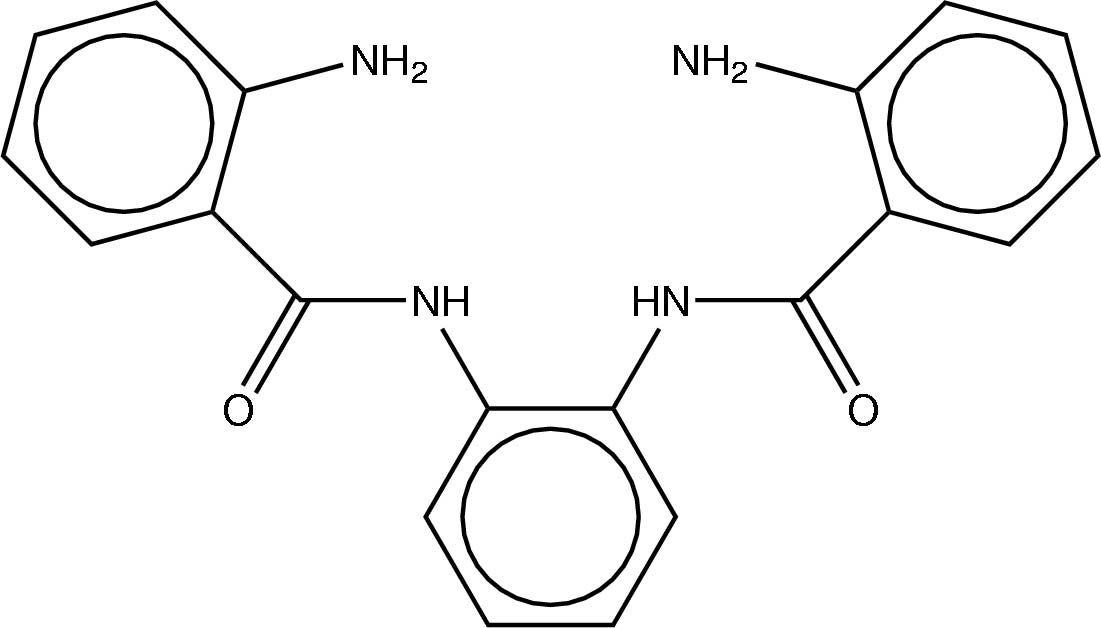


## Experimental

### 

#### Crystal data

C_20_H_18_N_4_O_2_
*M* *_r_* = 346.38
Monoclinic, 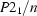

*a* = 8.7464 (3) Å
*b* = 14.4308 (6) Å
*c* = 13.6161 (6) Å
β = 97.291 (3)°
*V* = 1704.69 (12) Å^3^
*Z* = 4
Mo *K*α radiation
μ = 0.09 mm^−1^
*T* = 200 K
0.16 × 0.14 × 0.10 mm

#### Data collection

Nonius KappaCCD diffractometer
Absorption correction: none
7575 measured reflections
3893 independent reflections
2085 reflections with *I* > 2σ(*I*)
*R*_int_ = 0.049

#### Refinement

*R*[*F* ^2^ > 2σ(*F* ^2^)] = 0.047
*wR*(*F* ^2^) = 0.122
*S* = 0.99
3893 reflections
308 parameters
All H-atom parameters refined
Δρ_max_ = 0.19 e Å^−3^
Δρ_min_ = −0.20 e Å^−3^

### 

Data collection: *COLLECT* (Nonius, 2004 ▶); cell refinement: *SCALEPACK* (Otwinowski & Minor, 1997 ▶); data reduction: *DENZO* (Otwinowski & Minor, 1997 ▶) and *SCALEPACK*; program(s) used to solve structure: *SIR97* (Altomare *et al.*, 1999 ▶); program(s) used to refine structure: *SHELXL97* (Sheldrick, 2008 ▶); molecular graphics: *ORTEP-3* (Farrugia, 1997 ▶) and *Mercury* (Macrae *et al.*, 2006 ▶); software used to prepare material for publication: *PLATON* (Spek, 2009 ▶) and *WinGX* (Farrugia, 1999 ▶).

## Supplementary Material

Crystal structure: contains datablocks I, global. DOI: 10.1107/S1600536809009283/sj2594sup1.cif

Structure factors: contains datablocks I. DOI: 10.1107/S1600536809009283/sj2594Isup2.hkl

Additional supplementary materials:  crystallographic information; 3D view; checkCIF report

## Figures and Tables

**Table 1:** Hydrogen-bond geometry (Å, °)

*D*—H⋯*A*	*D*—H	H⋯*A*	*D*⋯*A*	*D*—H⋯*A*
N1—H1⋯O2	0.849 (17)	1.986 (18)	2.694 (2)	140.4 (17)
N3—H3⋯O1^i^	0.86 (2)	2.10 (2)	2.929 (2)	163.0 (17)
N4—H42⋯O2	0.97 (3)	1.88 (3)	2.646 (3)	134 (2)
N2—H21⋯O1	0.95 (2)	1.95 (2)	2.667 (2)	130.2 (19)

Symmetry code: (i) 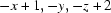.

**Fig. 1.**
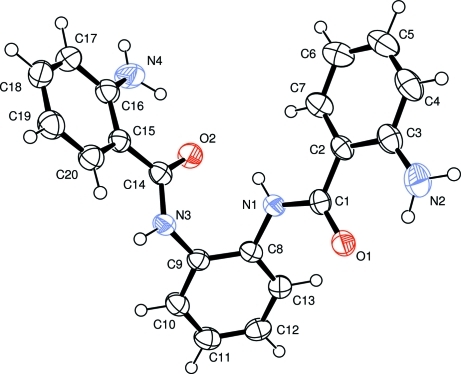
The molecular structure of the title compound (anisotropic displacement ellipsoids drawn at the 50% probability level).

**Fig. 2.**
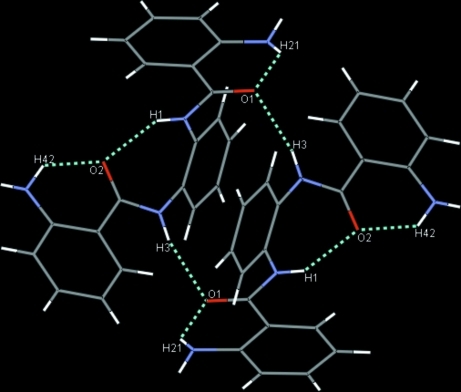
Hydrogen bonds (dashed lines) determining the conformational arrangement of the rings. For details of the hydrogen bonds see Table 1.

## supplementary crystallographic information

### Comment

In the present work the structure of *N*,*N*'-(1,2-phenylene)bis(2-aminobenzamide) has been determined to explore its suitability as a tetradentate ligand for various metal ions. The conformations of N—H and C═O bonds in the amide groups are *trans* to each other (Fig. 1), similar to that observed in other benzamides and benzanilides (Gowda *et al.*, 2003, 2008). Also, the conformations of the amide O atoms are *syn* to the *ortho* amino groups in the benzoyl rings. The amide group N1HC1O1 makes dihedral angles of 8.4 (2)° and 51.85 (16)° with the benzoyl and phenylene rings respectively. For the N3HC14O2 group, these values are 13.8 (2)° and 51.19 (17)°. The C2–C7 and C15–C20 benzoyl rings form dihedral angles of 59.64 (17)° and 64.86 (18)° respectively with the phenylene ring.

The conformational arrangement of the rings is mainly determined by intra- and intermolecular hydrogen-bonds. The graph set descriptor for the intramolecular hydrogen bonds is S(6)S(6)S(7) on a unitary level. Centrosymmetric dimers are formed by two intermolecular hydrogen bonds of the type N—H···O resulting in a *R*^2^_2_(14) descriptor on a unitary level. The hydrogen bonding pattern is shown in Fig. 2.

### Experimental

The title compound was prepared according to the literature method (Black & Rothnie, 1983). The purity of the compound was checked by determining its melting point. It was characterized by recording its IR and ^1^H NMR spectra. Single crystals of the title compound were obtained from a pyridine/ethanol (1:1, v/v) solution.

### Refinement

The H atoms were located in the difference map, their positional and isotropic vibrational parameters were refined freely.

### Crystal data

**Table:**

C_20_H_18_N_4_O_2_	*F*(000) = 728
*M**_r_* = 346.38	*D*_x_ = 1.350 Mg m^−^^3^
Monoclinic, *P*2_1_/*n*	Melting point: 532 K
Hall symbol: -P 2yn	Mo *K*α radiation, λ = 0.71073 Å
*a* = 8.7464 (3) Å	Cell parameters from 12612 reflections
*b* = 14.4308 (6) Å	θ = 3.1–27.5°
*c* = 13.6161 (6) Å	µ = 0.09 mm^−^^1^
β = 97.291 (3)°	*T* = 200 K
*V* = 1704.69 (12) Å^3^	Block, brown
*Z* = 4	0.16 × 0.14 × 0.10 mm

### Data collection

**Table:**

Nonius KappaCCD diffractometer	2085 reflections with *I* > 2σ(*I*)
Radiation source: rotating anode	*R*_int_ = 0.049
MONTEL, graded multilayered X-ray optics	θ_max_ = 27.5°, θ_min_ = 3.2°
Detector resolution: 9 pixels mm^-1^	*h* = −11→11
CCD; rotation images; thick slices scans	*k* = −18→18
7575 measured reflections	*l* = −17→17
3893 independent reflections	

### Refinement

**Table:**

Refinement on *F*^2^	Secondary atom site location: difference Fourier map
Least-squares matrix: full	Hydrogen site location: inferred from neighbouring sites
*R*[*F*^2^ > 2σ(*F*^2^)] = 0.047	All H-atom parameters refined
*wR*(*F*^2^) = 0.122	*w* = 1/[σ^2^(*F*_o_^2^) + (0.0521*P*)^2^] where *P* = (*F*_o_^2^ + 2*F*_c_^2^)/3
*S* = 0.99	(Δ/σ)_max_ = 0.001
3893 reflections	Δρ_max_ = 0.19 e Å^−^^3^
308 parameters	Δρ_min_ = −0.19 e Å^−^^3^
0 restraints	Extinction correction: *SHELXL97* (Sheldrick, 2008)
Primary atom site location: structure-invariant direct methods	Extinction coefficient: 0.0130 (17)

### Fractional atomic coordinates and isotropic or equivalent isotropic displacement parameters (Å^2^)

**Table:**

	*x*	*y*	*z*	*U*_iso_*/*U*_eq_	
O1	0.50757 (13)	0.19967 (8)	1.10126 (9)	0.0426 (4)	
O2	0.33370 (13)	0.11696 (9)	0.75452 (9)	0.0431 (4)	
N1	0.45030 (17)	0.17105 (11)	0.93824 (12)	0.0371 (4)	
N2	0.3038 (2)	0.28860 (15)	1.19702 (14)	0.0599 (5)	
N3	0.43678 (16)	−0.00594 (12)	0.84087 (11)	0.0364 (4)	
N4	0.1025 (3)	0.08932 (14)	0.61238 (15)	0.0579 (5)	
C1	0.41993 (19)	0.21187 (12)	1.02250 (14)	0.0343 (4)	
C2	0.27907 (18)	0.27011 (11)	1.01692 (13)	0.0350 (4)	
C3	0.2292 (2)	0.30615 (13)	1.10429 (15)	0.0437 (5)	
C4	0.0941 (2)	0.35939 (15)	1.0947 (2)	0.0552 (6)	
C5	0.0119 (2)	0.37821 (15)	1.00496 (19)	0.0566 (6)	
C6	0.0630 (2)	0.34564 (15)	0.91861 (18)	0.0552 (6)	
C7	0.1946 (2)	0.29271 (14)	0.92620 (16)	0.0457 (5)	
C8	0.58760 (18)	0.12237 (12)	0.92527 (12)	0.0334 (4)	
C9	0.57907 (18)	0.03780 (12)	0.87594 (12)	0.0331 (4)	
C10	0.7152 (2)	−0.00822 (14)	0.86314 (14)	0.0408 (5)	
C11	0.8571 (2)	0.02948 (15)	0.89720 (14)	0.0451 (5)	
C12	0.8644 (2)	0.11444 (14)	0.94410 (14)	0.0422 (5)	
C13	0.7312 (2)	0.16123 (14)	0.95732 (13)	0.0377 (5)	
C14	0.32233 (19)	0.03407 (13)	0.77899 (12)	0.0346 (4)	
C15	0.18663 (18)	−0.02232 (12)	0.74265 (12)	0.0339 (4)	
C16	0.0793 (2)	0.00956 (13)	0.66357 (13)	0.0419 (5)	
C17	−0.0521 (2)	−0.04422 (16)	0.63397 (16)	0.0501 (5)	
C18	−0.0768 (2)	−0.12620 (16)	0.67972 (17)	0.0515 (6)	
C19	0.0285 (2)	−0.15877 (15)	0.75605 (16)	0.0480 (5)	
C20	0.1582 (2)	−0.10711 (13)	0.78674 (15)	0.0408 (5)	
H13	0.7350 (18)	0.2213 (13)	0.9913 (13)	0.043 (5)*	
H20	0.2287 (19)	−0.1285 (12)	0.8428 (13)	0.044 (5)*	
H1	0.3885 (19)	0.1773 (13)	0.8853 (13)	0.040 (6)*	
H10	0.7102 (18)	−0.0671 (13)	0.8292 (12)	0.041 (5)*	
H12	0.960 (2)	0.1435 (12)	0.9685 (13)	0.047 (5)*	
H3	0.435 (2)	−0.0647 (15)	0.8504 (13)	0.044 (6)*	
H11	0.951 (2)	−0.0045 (13)	0.8853 (15)	0.062 (6)*	
H5	−0.083 (2)	0.4151 (14)	1.0036 (13)	0.059 (6)*	
H17	−0.132 (2)	−0.0200 (13)	0.5784 (14)	0.056 (6)*	
H18	−0.168 (2)	−0.1625 (14)	0.6568 (14)	0.056 (6)*	
H7	0.231 (2)	0.2720 (13)	0.8671 (15)	0.050 (5)*	
H4	0.063 (2)	0.3824 (14)	1.1515 (16)	0.068 (7)*	
H19	0.011 (2)	−0.2158 (14)	0.7871 (13)	0.052 (6)*	
H41	0.027 (3)	0.1085 (16)	0.5717 (18)	0.076 (8)*	
H6	0.010 (2)	0.3612 (14)	0.8493 (17)	0.076 (7)*	
H42	0.178 (3)	0.1311 (17)	0.647 (2)	0.090 (8)*	
H21	0.405 (3)	0.2631 (16)	1.2012 (17)	0.083 (8)*	
H22	0.288 (3)	0.3284 (19)	1.2441 (19)	0.090 (9)*	

### Atomic displacement parameters (Å^2^)

**Table:**

	*U*^11^	*U*^22^	*U*^33^	*U*^12^	*U*^13^	*U*^23^
O1	0.0509 (8)	0.0387 (8)	0.0375 (8)	0.0062 (6)	0.0034 (6)	−0.0021 (6)
O2	0.0499 (8)	0.0374 (8)	0.0412 (8)	−0.0016 (6)	0.0027 (6)	0.0061 (6)
N1	0.0371 (9)	0.0387 (10)	0.0349 (10)	0.0081 (7)	0.0030 (7)	−0.0038 (8)
N2	0.0682 (14)	0.0678 (14)	0.0466 (12)	0.0046 (10)	0.0183 (10)	−0.0122 (10)
N3	0.0359 (9)	0.0300 (10)	0.0427 (10)	0.0037 (7)	0.0025 (7)	0.0016 (8)
N4	0.0608 (12)	0.0560 (13)	0.0524 (12)	0.0074 (10)	−0.0109 (10)	0.0137 (10)
C1	0.0402 (10)	0.0275 (10)	0.0362 (11)	−0.0034 (8)	0.0095 (9)	0.0002 (8)
C2	0.0378 (10)	0.0266 (10)	0.0423 (11)	0.0012 (8)	0.0113 (8)	0.0010 (8)
C3	0.0472 (11)	0.0365 (12)	0.0508 (13)	−0.0022 (9)	0.0190 (10)	−0.0027 (9)
C4	0.0543 (13)	0.0481 (14)	0.0689 (17)	0.0041 (11)	0.0304 (13)	−0.0071 (12)
C5	0.0436 (12)	0.0419 (13)	0.0876 (19)	0.0088 (10)	0.0206 (13)	0.0030 (12)
C6	0.0478 (12)	0.0506 (14)	0.0675 (16)	0.0117 (10)	0.0088 (11)	0.0100 (12)
C7	0.0450 (11)	0.0459 (13)	0.0481 (13)	0.0087 (9)	0.0135 (10)	0.0040 (10)
C8	0.0332 (9)	0.0344 (11)	0.0333 (10)	0.0045 (8)	0.0072 (7)	0.0033 (8)
C9	0.0343 (10)	0.0332 (11)	0.0318 (10)	0.0030 (8)	0.0048 (7)	0.0029 (8)
C10	0.0428 (11)	0.0397 (12)	0.0410 (12)	0.0073 (9)	0.0089 (9)	−0.0009 (9)
C11	0.0380 (11)	0.0521 (14)	0.0462 (13)	0.0092 (10)	0.0093 (9)	0.0028 (10)
C12	0.0336 (11)	0.0521 (14)	0.0409 (12)	−0.0017 (10)	0.0043 (9)	0.0058 (10)
C13	0.0400 (11)	0.0370 (12)	0.0364 (11)	−0.0004 (9)	0.0054 (8)	0.0022 (9)
C14	0.0388 (10)	0.0367 (12)	0.0302 (10)	0.0053 (9)	0.0114 (8)	0.0008 (9)
C15	0.0363 (10)	0.0355 (11)	0.0303 (10)	0.0035 (8)	0.0061 (8)	−0.0022 (8)
C16	0.0456 (11)	0.0428 (13)	0.0370 (12)	0.0075 (9)	0.0041 (9)	−0.0029 (9)
C17	0.0438 (12)	0.0540 (15)	0.0498 (14)	0.0046 (11)	−0.0045 (10)	−0.0093 (11)
C18	0.0405 (12)	0.0522 (15)	0.0613 (15)	−0.0014 (11)	0.0038 (10)	−0.0189 (12)
C19	0.0451 (12)	0.0421 (13)	0.0580 (14)	−0.0019 (10)	0.0113 (10)	−0.0032 (11)
C20	0.0406 (11)	0.0416 (12)	0.0403 (12)	0.0018 (9)	0.0055 (9)	−0.0002 (9)

### Geometric parameters (Å, °)

**Table:**

O1—C1	1.249 (2)	C6—H6	1.02 (2)
O2—C14	1.249 (2)	C7—H7	0.95 (2)
N1—C1	1.346 (2)	C8—C9	1.390 (2)
N1—C8	1.421 (2)	C8—C13	1.394 (2)
N1—H1	0.849 (17)	C9—C10	1.394 (2)
N2—C3	1.369 (3)	C10—C11	1.380 (3)
N2—H21	0.95 (2)	C10—H10	0.966 (19)
N2—H22	0.89 (3)	C11—C12	1.380 (3)
N3—C14	1.353 (2)	C11—H11	0.986 (19)
N3—C9	1.423 (2)	C12—C13	1.378 (3)
N3—H3	0.86 (2)	C12—H12	0.957 (18)
N4—C16	1.374 (2)	C13—H13	0.981 (18)
N4—H41	0.85 (2)	C14—C15	1.472 (2)
N4—H42	0.97 (3)	C15—C20	1.399 (2)
C1—C2	1.485 (2)	C15—C16	1.413 (2)
C2—C7	1.395 (3)	C16—C17	1.403 (3)
C2—C3	1.417 (2)	C17—C18	1.367 (3)
C3—C4	1.402 (3)	C17—H17	1.024 (19)
C4—C5	1.364 (3)	C18—C19	1.380 (3)
C4—H4	0.91 (2)	C18—H18	0.97 (2)
C5—C6	1.391 (3)	C19—C20	1.378 (3)
C5—H5	0.98 (2)	C19—H19	0.945 (19)
C6—C7	1.374 (3)	C20—H20	0.968 (18)
			
C1—N1—C8	125.70 (16)	C8—C9—C10	118.97 (16)
C1—N1—H1	120.3 (12)	C8—C9—N3	122.85 (14)
C8—N1—H1	113.8 (12)	C10—C9—N3	118.14 (17)
C3—N2—H21	117.2 (14)	C11—C10—C9	121.07 (19)
C3—N2—H22	116.6 (16)	C11—C10—H10	119.5 (10)
H21—N2—H22	116 (2)	C9—C10—H10	119.4 (10)
C14—N3—C9	124.48 (17)	C10—C11—C12	119.55 (18)
C14—N3—H3	119.2 (12)	C10—C11—H11	118.6 (11)
C9—N3—H3	114.9 (12)	C12—C11—H11	121.8 (11)
C16—N4—H41	116.7 (15)	C13—C12—C11	120.32 (18)
C16—N4—H42	114.2 (15)	C13—C12—H12	117.2 (11)
H41—N4—H42	122 (2)	C11—C12—H12	122.5 (11)
O1—C1—N1	120.28 (16)	C12—C13—C8	120.35 (19)
O1—C1—C2	122.57 (16)	C12—C13—H13	121.0 (10)
N1—C1—C2	117.15 (16)	C8—C13—H13	118.6 (10)
C7—C2—C3	118.21 (17)	O2—C14—N3	119.81 (16)
C7—C2—C1	121.35 (16)	O2—C14—C15	121.86 (16)
C3—C2—C1	120.42 (16)	N3—C14—C15	118.33 (17)
N2—C3—C4	118.99 (19)	C20—C15—C16	118.35 (17)
N2—C3—C2	123.05 (17)	C20—C15—C14	121.25 (16)
C4—C3—C2	117.92 (19)	C16—C15—C14	120.38 (17)
C5—C4—C3	122.4 (2)	N4—C16—C17	119.08 (19)
C5—C4—H4	120.6 (13)	N4—C16—C15	122.20 (18)
C3—C4—H4	117.0 (13)	C17—C16—C15	118.67 (19)
C4—C5—C6	120.0 (2)	C18—C17—C16	121.3 (2)
C4—C5—H5	118.2 (11)	C18—C17—H17	120.0 (10)
C6—C5—H5	121.8 (11)	C16—C17—H17	118.7 (10)
C7—C6—C5	118.7 (2)	C17—C18—C19	120.6 (2)
C7—C6—H6	118.2 (12)	C17—C18—H18	119.4 (11)
C5—C6—H6	123.1 (12)	C19—C18—H18	120.0 (11)
C6—C7—C2	122.8 (2)	C20—C19—C18	119.3 (2)
C6—C7—H7	118.4 (11)	C20—C19—H19	120.4 (11)
C2—C7—H7	118.8 (11)	C18—C19—H19	120.3 (11)
C9—C8—C13	119.69 (15)	C19—C20—C15	121.82 (19)
C9—C8—N1	119.96 (15)	C19—C20—H20	118.9 (10)
C13—C8—N1	120.27 (16)	C15—C20—H20	119.1 (10)
			
C8—N1—C1—O1	−8.7 (3)	C8—C9—C10—C11	−1.0 (3)
C8—N1—C1—C2	171.86 (15)	N3—C9—C10—C11	−178.69 (17)
O1—C1—C2—C7	171.32 (17)	C9—C10—C11—C12	−0.5 (3)
N1—C1—C2—C7	−9.2 (2)	C10—C11—C12—C13	0.3 (3)
O1—C1—C2—C3	−7.1 (3)	C11—C12—C13—C8	1.3 (3)
N1—C1—C2—C3	172.34 (16)	C9—C8—C13—C12	−2.8 (3)
C7—C2—C3—N2	−179.81 (18)	N1—C8—C13—C12	−179.49 (16)
C1—C2—C3—N2	−1.3 (3)	C9—N3—C14—O2	−5.0 (2)
C7—C2—C3—C4	2.6 (3)	C9—N3—C14—C15	175.18 (14)
C1—C2—C3—C4	−178.97 (16)	O2—C14—C15—C20	−165.42 (16)
N2—C3—C4—C5	−178.67 (19)	N3—C14—C15—C20	14.4 (2)
C2—C3—C4—C5	−0.9 (3)	O2—C14—C15—C16	12.8 (2)
C3—C4—C5—C6	−1.2 (3)	N3—C14—C15—C16	−167.37 (15)
C4—C5—C6—C7	1.6 (3)	C20—C15—C16—N4	−176.21 (16)
C5—C6—C7—C2	0.1 (3)	C14—C15—C16—N4	5.5 (3)
C3—C2—C7—C6	−2.2 (3)	C20—C15—C16—C17	1.1 (3)
C1—C2—C7—C6	179.34 (17)	C14—C15—C16—C17	−177.18 (14)
C1—N1—C8—C9	135.32 (18)	N4—C16—C17—C18	177.07 (18)
C1—N1—C8—C13	−48.0 (3)	C15—C16—C17—C18	−0.3 (3)
C13—C8—C9—C10	2.6 (2)	C16—C17—C18—C19	−0.7 (3)
N1—C8—C9—C10	179.31 (16)	C17—C18—C19—C20	0.8 (3)
C13—C8—C9—N3	−179.83 (16)	C18—C19—C20—C15	0.0 (3)
N1—C8—C9—N3	−3.1 (2)	C16—C15—C20—C19	−1.0 (3)
C14—N3—C9—C8	55.6 (2)	C14—C15—C20—C19	177.33 (16)
C14—N3—C9—C10	−126.74 (18)		

### Hydrogen-bond geometry (Å, °)

**Table:**

*D*—H···*A*	*D*—H	H···*A*	*D*···*A*	*D*—H···*A*
N1—H1···O2	0.849 (17)	1.986 (18)	2.694 (2)	140.4 (17)
N3—H3···O1^i^	0.86 (2)	2.10 (2)	2.929 (2)	163.0 (17)
N4—H42···O2	0.97 (3)	1.88 (3)	2.646 (3)	134 (2)
N2—H21···O1	0.95 (2)	1.95 (2)	2.667 (2)	130.2 (19)

Symmetry codes: (i) −*x*+1, −*y*, −*z*+2.
